# Medical Students’ Confidence After “CardioSim”: A Low-Fidelity, Peer Role-Play Simulation

**DOI:** 10.7759/cureus.67192

**Published:** 2024-08-19

**Authors:** Nichola Philp, Asmaa Omran, Michael J Otorkpa, Alan Kirk

**Affiliations:** 1 Cardiothoracic Surgery, Golden Jubilee National Hospital, Glasgow, GBR; 2 Thoracic Surgery, Golden Jubilee National Hospital, Glasgow, GBR

**Keywords:** peer role play, low fidelity, medical education, simulation, simulation in medical education, cardiology

## Abstract

Introduction: Plans to increase medical student numbers will increase costs and potentially reduce clinical exposure. Simulation can be utilised to fill that gap. Low-fidelity simulation with peer role-play (PRP) provides reduced costs and standardisation of experience compared to high-fidelity or simulated patient RP simulation. This study aimed to assess changes in confidence in common graduate-level skills following a low-fidelity PRP scenario.

Methods: Forty-three fourth-year medical students were divided into groups of three and five students. A Likert-scale questionnaire was completed at the start of the session. During the simulation, they had the opportunity to be the doctor and the patient and observe the remaining scenarios. Finally, the same questionnaire was answered.

Results: All students gained confidence in at least two aspects. All students were confident in history taking and ST-elevation myocardial infarction (STEMI) management at the end of the session. The biggest improvement in overall confidence was in the management of non-ST-elevation myocardial infarction (NSTEMI) (62.8%) and Situation-Background-Assessment-Recommendation (SBAR) handover (60.5%). Six students improved their confidence by at least one ranking in every question, and one student improved their confidence by three rankings in four questions. SBAR handover and management of pneumothorax had the biggest improvements.

Conclusion: Low-fidelity RP simulation allows the development of confidence in technical and non-technical skills. Given increasing student numbers and increasing medical education costs, it can be utilised effectively as part of a simulation syllabus that helps prepare students for clinical practice. Areas of further research include focus groups, knowledge tests and comparisons to other forms of simulation.

## Introduction

To meet the growing needs of patients and to reduce pressures within the workforce, the Medical Schools Council has recommended increasing the number of graduates by 5,000, resulting in an average year group size of 200-250 students [1]. With the average cost of educating a medical student at £200,000, this will require an additional £1 billion of funding [1]. A proportion of this funding needs to be utilised to allow for equitable clinical experience for all students and include ways to support clinical education such as online resources or embedding students in a clinical team [1]. This could also be achieved by simulation.

Simulation refers to an imitation of a real-world situation that allows learners to practice for a situation and improve their abilities without risks of patient harm [2-4]. It can cover any educational activity including the use of peer role-play (PRP), simulated or standardized patients (SPs), or manikins and allows for the practice of communication and technical and non-technical skills [2,3,5]. There is a spectrum of high fidelity to low fidelity related to the degree of realism in the simulation. High-fidelity simulations are generally resource-intensive and expensive, not only considering the purchasing and maintenance of the equipment but also the faculty and their training requirements [5].

Manikins come in a range of fidelity. Low-fidelity manikins are usually static with limited capabilities and may only be a part of the body eg a plastic arm for cannulation practice [5,6]. High-fidelity manikins encompass the whole body and use a computer to demonstrate realistic physiology that reacts to the learners’ intervention. This allows the learner to develop non-technical skills such as decision-making, problem-solving, prioritisation and teamwork [5,6].

SP also have various fidelity depending on what is being simulated, with some studies classifying them as high fidelity and others considering them low fidelity. Arguments for them to be high fidelity include the expense, including hiring the person and their time-consuming training, and the high degree of realism that can be achieved when used in communication skills simulations [7,8]. However, when compared to high-fidelity manikins, SPs are often low fidelity for simulating clinical signs [9,10]. They may also be considered low fidelity if the scenarios are oversimplified without any major challenges [11]. The advantages of using SP are that they provide uniformity in the scenarios for multiple students and they are trained in providing feedback to the learners [7,12].

Alternatively, PRP involves the students taking turns playing both the doctor and the patient and is therefore an easily implemented, low-cost tool [7-9,13]. It provides experiential learning to both students involved, allowing for increased understanding of patient perspectives and a more empathetic approach [7-10,13]. Due to the availability of participants, an increased number and variety of clinical scenarios can be covered whilst ensuring homogeneity between student groups [13]. However, to be effective, the sessions need to be well designed and the tutors trained appropriately [7,9,14].

This study aimed to assess student’s confidence in common skills required at the graduate level following a low-fidelity PRP scenario. The hypothesis is that students would gain benefit in at least one of the skills assessed and this would be irrespective of the simulation scenario they completed.

## Materials and methods

Fourth-year medical students at the University of Glasgow, Scotland, in their cardiology clinical block were divided into groups of three and five students to complete the “CardioSim”: a low-fidelity PRP simulation (N = 43). During the session, the “patient” is given a brief scenario with the salient clinical features but can add in their own knowledge of the presentation and have the option to be creative with aspects of their social history. The tutor will correct or add any important features that have been forgotten.

The session covers communication skills of history taking and Situation-Background-Assessment-Recommendation (SBAR) handover, ABCDE (A-E) assessment with students asking for findings that are provided by the tutor and clinical reasoning skills of investigation, diagnosis and management. The patients then all deteriorate allowing for repeated practice. The session is designed to consolidate learning from junior years of medical school and prepare students for the first years of clinical practice.

Each session is run by two tutors (NP, AO or MO). There are nine available scenarios, including two core scenarios that are run every session (non-ST-elevation myocardial infarction (NSTEMI) deteriorating to ST-elevation myocardial infarction (STEMI) and pneumothorax deteriorating to tension pneumothorax). Each student got the opportunity to be both a doctor and a patient and they observed the other scenarios. Each session follows the same general structure but can be flexible depending on questions from students.

A five-point Likert-scale questionnaire (Table 1) was designed to assess student confidence in skills that would be covered regardless of the scenario in which the student played the doctor. A Likert scale was selected as there was ease of implementation and interpretation whilst still providing some nuance in response to ensure validity and reliability in the results. The students were asked to rate how confident they felt with the following skills on a scale of Unconfident, Slightly unconfident, Neither confident nor unconfident, Slightly confident or Confident. The skills assessed were history taking, A-E assessment, electrocardiogram (ECG) interpretation, arterial blood gas (ABG) interpretation, chest X-ray (CXR) interpretation, management of NSTEMI, management of STEMI, management of pneumothorax and SBAR handover. Only the core scenarios were assessed to allow flexibility in the other scenarios to cover various aspects of the curriculum. A multiple-choice questionnaire was considered, but this was felt to be time-consuming and might give the students the answers prior to the sessions.

**Table 1:** Questions asked to students scored using a five-point Likert scale: Unconfident, Slightly unconfident, Neither confident nor unconfident, Slightly confident, or Confident A-E = ABCDE, ECG = electrocardiogram, ABG = arterial blood gas, CXR = chest X-ray, NSTEMI = non-ST-elevation myocardial infarction, STEMI = ST-elevation myocardial infarction, SBAR = Situation-Background-Assessment-Recommendation

Likert-scale questionnaire - How confident do you feel with the following?					
	Unconfident	Slightly unconfident	Neither confident nor unconfident	Slightly confident	Confident
History taking					
A-E assessment					
ECG interpretation					
ABG interpretation					
CXR interpretation					
Management of NSTEMI					
Management of STEMI					
Management of pneumothorax					
SBAR handover					

All students were invited to participate in the survey after an explanation from the tutors. The session would continue regardless of whether they completed the questionnaire, so there was no impact on their learning if they declined to participate. Ethical approval was not required as students gave informed consent and there was no impact on their education. Students were excluded from the study if they were late and missed part of the first scenario as this would be one of the two core scenarios.

Pre-test questionnaires were completed with pen and paper after an introduction to the simulation and an explanation of the study. All the students then participated in the simulation. At the end of all the scenarios, the students were then asked to complete the post-test questionnaire. Both questionnaires were then collected by the tutors.

Data were recorded and analysed with simple statistics in Microsoft Excel and Microsoft Forms (Microsoft Corporation, USA).

## Results

Forty-three students completed the questionnaire. Only 13 students completed the gender demographic question (six male and seven female), and only 11 stated whether they were undergraduate or postgraduate, used as a surrogate marker for age. All 11 were undergraduates. One student recorded their scenario; they completed the core pneumothorax scenario. There was one piece of missing data as one student did not complete the pre-test question on the management of pneumothorax.

**Figure 1:**
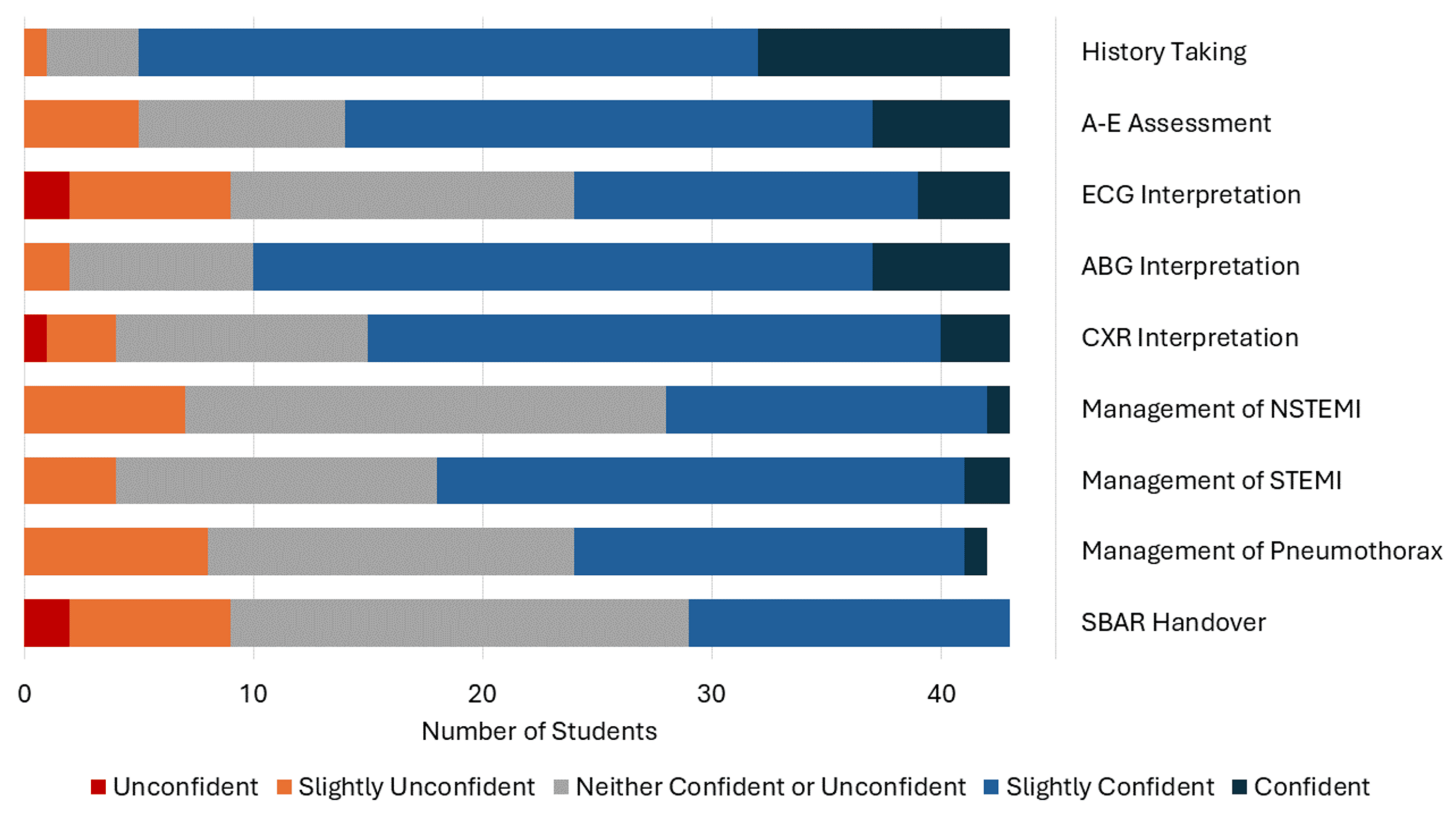
Results of the Likert-scale questionnaire before the simulation (pre-test) Number of students who answered each question: Unconfident (red), Slightly unconfident (orange), Neither confident nor unconfident (grey), Slightly confident (light blue) and Confident (dark blue). A-E = ABCDE, ECG = electrocardiogram, ABG = arterial blood gas, CXR = chest X-ray, NSTEMI = non-ST-elevation myocardial infarction, STEMI = ST-elevation myocardial infarction, SBAR = Situation-Background-Assessment-Recommendation

**Figure 2:**
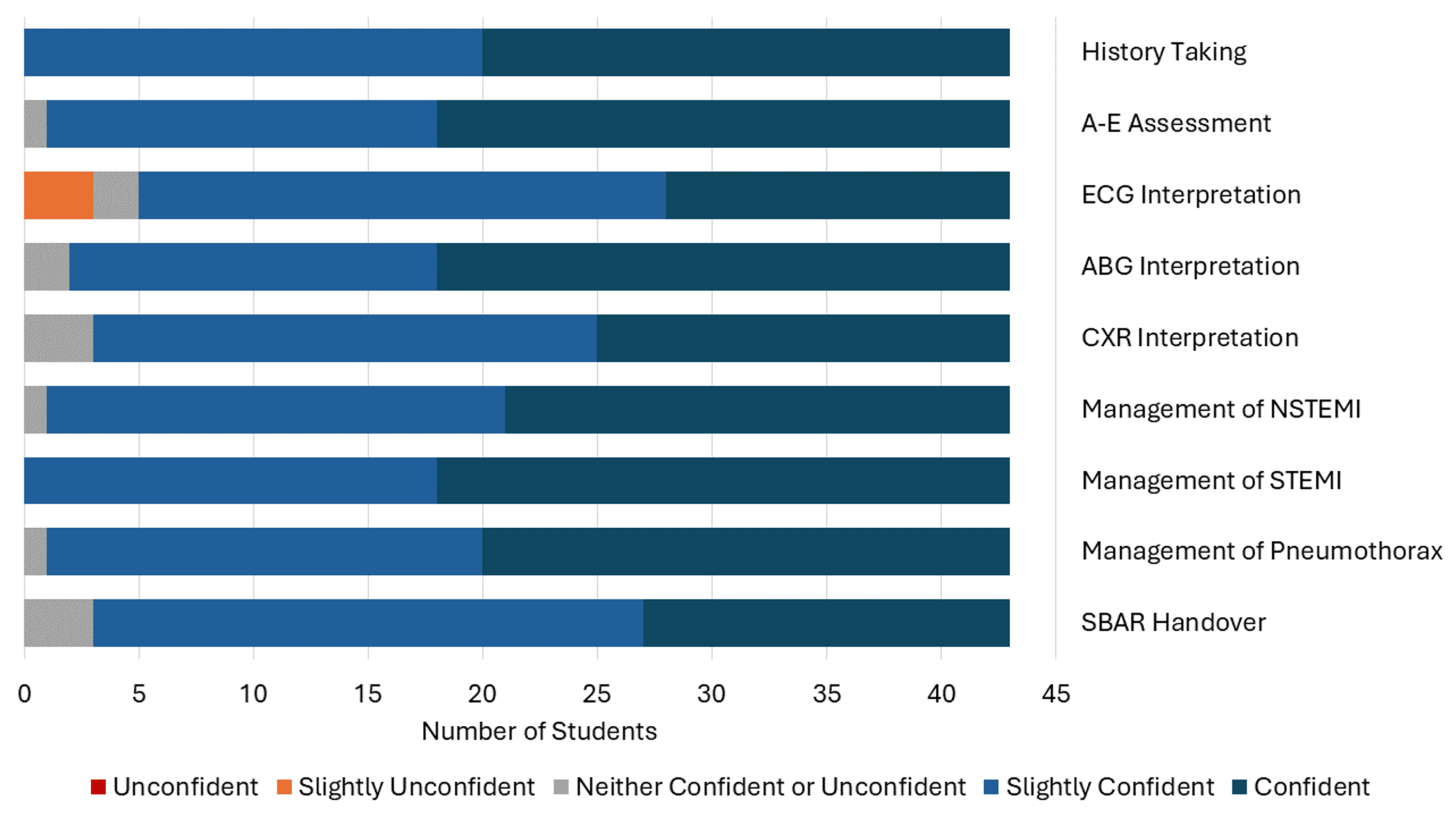
Results of the Likert-scale questionnaire after the simulation (post-test) Number of students who answered each question: Unconfident (red), Slightly unconfident (orange), Neither confident nor unconfident (grey), Slightly confident (light blue) and Confident (dark blue). A-E = ABCDE, ECG = electrocardiogram, ABG = arterial blood gas, CXR = chest X-ray, NSTEMI = non-ST-elevation myocardial infarction, STEMI = ST-elevation myocardial infarction, SBAR = Situation-Background-Assessment-Recommendation

Overall, the results were positive with all students gaining confidence in at least two aspects. Thirty-eight students were confident or slightly confident in history taking in the pretest, and this increased to 43 in the post-test. For the A-E assessment, these numbers were 29 in the pretest to 42 in the post-test; ECG interpretation went from 19 to 38; ABG interpretation from 33 to 41; CXR interpretation 28 to 40; management of NSTEMI 15 to 42; management of STEMI 25 to 43; management of pneumothorax 18 to 42; and SBAR handover 14 to 40. All students now feel confident in history taking and STEMI management. The biggest improvement in overall confidence was in the management of NSTEMI and SBAR handover, which had 62.8% and 60.5% increases, respectively. The full pretest results are presented in Figure 1, and the full post-test results are in Figure 2.

The most common improvement was to move up one ranking (53.4%), and this occurred most commonly in the SBAR handover (28 students). SBAR handover and management of pneumothorax each had three students who improved their confidence up to three rankings. Six students improved their confidence by at least one ranking in every question. One student improved their confidence by only one ranking but in all questions; one student improved their confidence by two rankings in eight questions, and one student improved their confidence by three rankings in four questions.

The student who improved the least was unchanged in seven questions and only improved by one ranking in the other two questions. Overall, 27.2% remained unchanged in their confidence. The most common question where no change occurred was history taking, but this was the area with the highest pre-test confidence.

Unfortunately, three students reduced their confidence ranking in one question by one ranking: one in history taking, one in ECG interpretation and one in CXR interpretation. There is no further data to explain why this was the case. The students all improved in at least five other questions.

Some students left feedback comments on the simulation, which were generally positive and backed up the improvements in confidence seen in the questionnaire:

“CardioSim was great, no time wasting and lots of learning.”

“I thought it was much better than usual sim as we were guided to get everything correct eg in A-E and management plans.”

“It was good to do the acting as well as it made you think about how a patient would present with the various pathologies”

“All of the cardio simulation was very useful.”

“Great session, I preferred this format to the mannequin sim as I feel I learned more. I liked that the scenario kept going”

“Could maybe have some teaching on the scenarios which were not covered in the session”

## Discussion

High fidelity versus low fidelity

Multiple studies have looked at the impact of the fidelity of a simulation on both participants’ perspectives and their educational outcomes. Low-fidelity simulation was deemed valuable as it increased learners’ confidence and made them feel more knowledgeable and skilful when compared to no other training [3,15]. When compared to high-fidelity manikins, some learners prefer the lower-fidelity simulations, but this could be related to their familiarity with the style of learning [5].

In terms of the educational impact of low-fidelity simulation, it has been shown to objectively improve knowledge and clinical skills when compared to no training [3,15]. It has also been shown to have validity in predicting overall job performance and as such can have applications in high-stakes selection processes [16]. In comparison to high-fidelity simulation, there has been no statistical difference in clinical performance [6,17]. However, students can place more value on high-fidelity simulation and anticipate better results; if these are not forthcoming, it can lead to overconfidence compared to those undertaking low-fidelity simulation who end up with more realistic evaluations of their own performances [17].

The choice of high-fidelity or low-fidelity simulation may depend on the stage of training of the participants and what level of competence is being demonstrated. Miller’s pyramid (knows, knows how, shows how, does) is a model of clinical competence progression from knowledge to action [17]. Generally, high-fidelity simulation allows for greater demonstration of the top levels of the pyramid (shows how and does) and is therefore more suitable for learners at a higher educational level [17,18]. However, as long as the simulation is adapted for the stage of training, students gain improved confidence from high- and low-fidelity simulation equally [5].

RP versus simulated/standardised patients (SP)

Multiple studies have looked at the impact of RP in simulation on both participants’ perspectives and their educational outcomes. Looking solely at RP, it is well liked by learners; deemed to be realistic, acceptable and effective; and improved confidence by 30%, and 87% of the participants felt that it improved their understanding [2,11,19,20]. When comparing RP to SP, both methods are liked by the participants and are deemed to be realistic, worthwhile, useful, challenging and applicable to training [7,12,14]. However, SPs were often preferred as they improved confidence and had a better perceived effect on training [2,7,12,14,21]. Improved confidence may be related to the active participation that both types of simulation encourage, providing the opportunity to practice their own communication skills and observe others [14].

In terms of educational effectiveness, RP alone produced significant improvement in communication skills, interpersonal skills and information delivery with scores of 9.4 out of 10 [11,13,19,22]. Comparing RP to SP, both groups display significantly improved communication skills, especially against controls, but with no significant difference between the types of simulation [2,12,21].

However, a study by Bosse et al. found that students had better self-efficacy ratings and better objective structured clinical examination (OSCE) scores following RP simulation over controls and SP simulation [9]. This is attributed to the fact that switching roles and being the patient increases empathy and understanding of the patient’s perspective. This was also seen in a study by Sasson et al. in which fourth-year students who acted as SPs for junior students went on to achieve better communication skills scores in their own assessments [23]. One student in our study noted this as a positive of the RP simulation.

A limitation of these studies is that they do not go on to investigate the long-term effectiveness of the learning. Kirkpatrick’s levels are a four-stage evaluation model that covers learners’ “reaction”, “learning”, “behaviour” and “results” following an educational intervention [2]. A 2020 systematic review found that studies focused on levels 1 and 2 with none of the 22 included studies covering “behaviour” with real patients or “results” for clinical populations and only one study exploring persistent improvement at six months [2].

Stress response and anxiety

Psychological impact can affect learners’ ability to retain information. Evidence has shown a positive impact if students are enjoying and engaged in the learning, but there is less research on whether the converse is true [17,22]. High-fidelity simulations can increase stress and anxiety, similar to the real clinical environment [4], and RP can induce feelings of shyness and awkwardness due to the prior relationship between the participants, giving the simulation a false nature [8,13].

Stress can be measured either subjectively or biologically. High-fidelity simulation causes greater stress pretraining but there was no statistical difference between pre- and post-training scores or between the simulation modalities [4,6,24]. Cognitive load scores are significantly higher with high-fidelity simulation, but this does not account for the high-fidelity simulation (managing an emergency situation) being a more complex task possibly requiring clinical reasoning, procedural and team management skills than the standard simulation (performing a spinal tap) [4].

Biological stress can be measured in various ways, including salivary cortisol levels and heart rate variability. Both high- and low-fidelity simulation raises cortisol levels; some studies suggest that high-fidelity simulation causes a significantly higher increase [4] but this is not universal [6]. Heart rate variability was found to increase significantly from baseline during the simulation regardless of the frequency of simulations, but there was no significant difference between the groups [25]. However, overall variability, and therefore stress, was lower in those that had more frequent simulations [25].

Despite studies showing an increase in stress, there is no consideration of how that relates to participants' performance or future clinical practice. Stress is important to improve performance as described by the Yerkes-Dodson law: an inverted-U curve where there is an optimal level of stress to obtain peak performance [25]. If stress levels go above or below the optimal, there will be a drop in performance. There is no evidence of whether the high levels of stress found in high-fidelity simulation create optimum stress or impair performance.

Cost-effectiveness

Cost-effectiveness is relative depending on what PRP is being compared to, and this depends on areas that the simulation is looking to develop. If the simulation is looking to develop communication skills, the PRP can be compared to the use of SP. The cost of hiring SP is variable but is around £200 per day [22]. This does not include the time and expense of training SP and the time spent by educators in the organisation [12,22]. Including training time means that SPs are 53.6% more expensive than PRP with a cost-effectiveness ratio of 0.36-0.45 compared to 0.74 with PRP [8,10]. In 1997, the costs of using SP for a class of 100 students were $2500, five times higher than PRP [21]. By 2017, the cost of SP was over $100 per student [26]. With inflation and the increasing number of students, an increased number of simulations need to be run and costs can quickly become prohibitive.

When assessing students’ skills in examination and managing an acutely unwell patient, a high-fidelity manikin is more realistic to clinical practice. The cost of the manikin is variable from £18000 to £44000 depending on the features [27,28]. This does not include the cost of the specific equipment needed to interact with the manikin, the simulation centre to house it and run the sessions, training for the users and any technological upgrades that will be required with time [28,29]. There will also be a limited number of manikins in any particular centre, and this needs to be shared amongst not just medical students but all health professions students, international graduates and external courses that may require the use of the simulation centre. This is the main reason why low-fidelity simulation is used in our teaching programme. Another consideration is that both the communication skills and the clinical assessment were only part of the skills assessed in this simulation, and therefore the use of both a manikin and SP would not be financially viable. As noted by the comments from the students in the current study, they value the variety and depths of topics covered, which would not be possible if trying to maximise the value of the manikin.

Limitations

There are limitations to the study design. Firstly, there was no control group as we did not have the facilities to run the simulation with either SPs or manikins, and we did not want to disadvantage a group of students by not undergoing the simulation. The research team ran the simulation and was in the room when the questionnaire was completed. Every effort was made to allow the students privacy to complete it, and it was only handed to the researchers at the end of the session, but students may have felt inclined to give more positive results.

Another limitation was the small sample size. This could be improved by extending the duration of the study to allow more students to complete the simulation. This would improve the validity and reliability of the results.

Most students declined to fill in the demographic data, and only one recorded which simulation they underwent, so it has not been possible to assess whether undertaking the core simulations has impacted their confidence in managing those conditions. There were also no data collected on what specific aspects the students found helpful or why they gave these results, particularly the three students who felt less confident in some aspects. This could be an area of future research by conducting focus groups with the students.

Another area of further research would be to evaluate any long-term impact of this session. As with much of the literature, it assesses Kirkpatrick level 1. A pre- and post-simulation knowledge test could be added, or the analysis of end-of-year examinations could be performed to assess higher levels. 

## Conclusions

Low-fidelity RP simulation plays an important role in the student curriculum as it allows the development of confidence in important technical and non-technical skills such as clinical reasoning, diagnosis and management. There are advantages and disadvantages compared to high-fidelity or simulated PRP depending on which skills are being developed. Given the increasing student numbers and increasing costs of medical education, it can be utilised effectively as part of a simulation syllabus that helps prepare students for clinical practice.

In terms of common graduate skills of history taking, A-E assessment, ECG interpretation, ABG interpretation, CXR interpretation, management of NSTEMI, management of STEMI, management of pneumothorax and SBAR handover, every student improved their confidence in at least two skills and at least one student improved in every skill. The most commonly improved skill was SBAR handover and management of pneumothorax, and the SBAR handover had the biggest individual improvement. All students became confident or slightly confident in history taking and management of STEMI by the end of the session. Therefore, RP simulation plays an important role in medical education, even allowing the improvement in technical skills without the use of expensive high-fidelity manikins. 

There are several areas for further research. Firstly, focus groups could be conducted to ascertain which aspects of the simulation influenced the results, particularly for those who felt less confident after the session. Another area of research would be to look at the impact on higher Kirkpatrick levels, either by adding a knowledge test or by analysing end-of-year examination results. Finally, a direct comparison between different types of simulations could determine whether one is more effective at improving student confidence and learning.
